# Antibody-dependent enhancement (ADE) of SARS-CoV-2 pseudoviral infection requires FcγRIIB and virus-antibody complex with bivalent interaction

**DOI:** 10.1038/s42003-022-03207-0

**Published:** 2022-03-24

**Authors:** Shuang Wang, Junchao Wang, Xiaojuan Yu, Wen Jiang, Shuo Chen, Rongjuan Wang, Mingzhu Wang, Shasha Jiao, Yingying Yang, Wenbo Wang, Huilin Chen, Ben Chen, Chunying Gu, Chuang Liu, An Wang, Min Wang, Gang Li, Cuicui Guo, Datao Liu, Jinchao Zhang, Min Zhang, Lan Wang, Xun Gui

**Affiliations:** 1 Mabwell (Shanghai) Bioscience Co., Ltd, Shanghai, 201210 China; 2 Beijing Kohnoor Science & Technology Co., Ltd, Beijing, 102206 China; 3 School of Life Sciences, Anhui University, Hefei, 230601 China; 4 Key Laboratory of the Ministry of Health for Research on Quality and Standardization of Biotech Products, National Institutes for Food and Drug Control, Beijing, 100050 China; 5 Ludwig Cancer Research, Nuffield Department of Medicine, University of Oxford, Oxford, OX37DQ United Kingdom

**Keywords:** SARS-CoV-2, Infectious diseases

## Abstract

Understanding the underlying molecular mechanisms behind ADE of SARS-CoV-2 is critical for development of safe and effective therapies. Here, we report that two neutralizing mAbs, MW01 and MW05, could enhance the infection of SARS-CoV-2 pseudovirus on FcγRIIB-expressing B cells. X-ray crystal structure determination and S trimer-binding modeling showed that MW01 and MW05 could bind to RBDs in S trimer with both “up” and “down” states. While, the neutralizing mAb MW07, which has no ADE activity only binds to RBD in S trimer with “up” state. Monovalent MW01 and MW05 completely diminished the ADE activity compared with their bivalent counterparts. Moreover, both macropinocytosis and endocytosis are confirmed involving in ADE of SARS-CoV-2 pseudoviral infection. Blocking endosome transportation and lysosome acidification could inhibit the ADE activity mediated by MW05. Together, our results identified a novel ADE mechanism of SARS-CoV-2 pseudovirus in vitro, FcγRIIB-mediated uptake of SARS-CoV-2/mAb complex with bivalent interaction.

## Abstract (web summary)

By distinguishing between two types of neutralizing antibodies that either exhibit or lack antibody-dependent enhancement (ADE) activity, FcγRIIB and bivalent binding are shown to be required for ADE in SARS-CoV-2 infection.

## Introduction

Coronavirus disease 2019 (COVID-19), caused by severe acute respiratory syndrome coronavirus-2 (SARS-CoV-2), has resulted in substantial global morbidity and mortality along with widespread social and economic disruption^1^. As of December 16, 2021, SARS-CoV-2 has spread to more than 200 countries and caused more than 5.3 million deaths (World Health Organization). Vaccines against SARS-CoV-2 are essential countermeasure to prevent the naïve population from acquiring COVID-19 disease, and several SARS-CoV-2 vaccine candidates developed by different platforms have been evaluated in preclinical models and clinical trials^2–6^. Although deployment of SARS-CoV-2 vaccines is the most effective strategy to control the COVID-19 pandemic and prevent the re-emergence of SARS-CoV-2, immediate therapeutics are also needed. Potent neutralizing monoclonal antibodies (mAbs) are a promising class of prophylactics and therapeutics against SARS-CoV-2 infection^7–10^. Administration of COVID-19 patients with potent neutralizing mAbs or convalescent plasma improved clinical outcomes and decreased viral loads, highlighting the immediate protection efficacy of neutralizing mAbs against SARS-CoV-2^11,12^.

Exploring the underlying mechanisms of SARS-CoV-2 neutralizing mAbs is critical for the development of therapeutics with effectiveness and safety. ADE of SARS-CoV-2 infection is a major concern for the development of antibody-based vaccines and therapeutics. ADE could increase the severity of several viral infections, including Respiratory Syncytial virus (RSV), Dengue virus, Zika virus, SARS-CoV and Middle East Respiratory Syndrome Coronavirus (MERS-CoV)^13–18^. Various hypotheses about the mechanisms of ADE triggered by different viruses were reported, such as Fc receptors-mediated enhancement of SARS-CoV infection, complement-mediated enhancement of HIV infection and functional mimicry-mediated MERS-CoV infection^19–21^. However, the detailed molecular mechanisms of ADE of SARS-CoV-2 infection are still largely unknown. We previously generated a panel of fully human SARS-CoV-2 neutralizing mAbs and observed the ADE activity for the lead mAb MW05, which allows us to explore the detailed ADE mechanisms for SARS-CoV-2 infection^22^.

## Results

### ADE of SARS-CoV-2 pseudoviral infection on Raji and Daudi cells mediated by MW01 and MW05

**Fig. 1:**
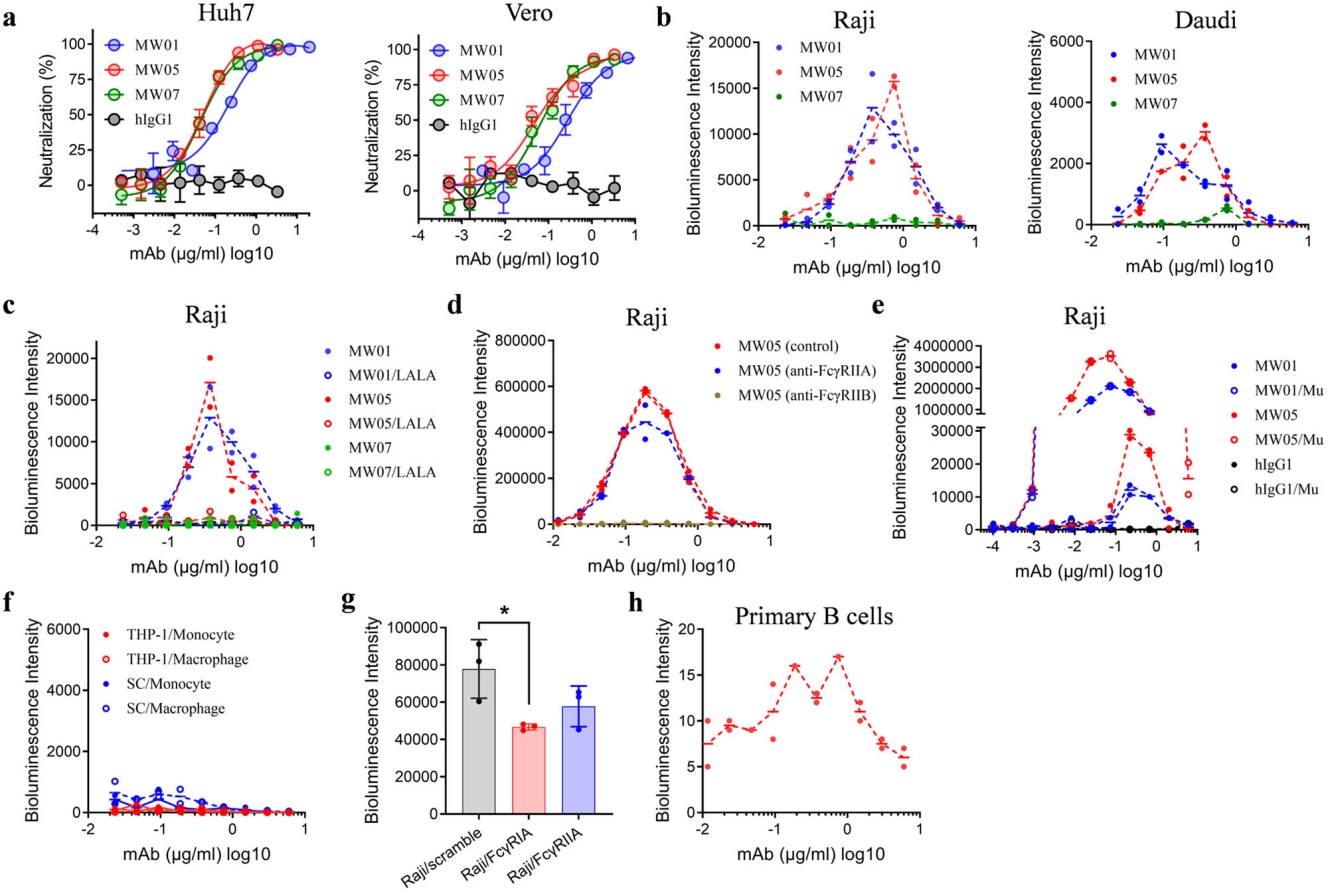
ADE activity of three SARS-CoV-2 neutralizing mAbs MW01, MW05 and MW07. **a** Neutralization potency of MW01, MW05 and MW07 against SARS-CoV-2 pseudovirus on Huh7 and Vero cells. SARS-CoV-2 pseudovirus were incubated with 3-fold serially diluted mAbs. The mixtures were then added into Huh7 or Vero cells. After 24 h incubation, neutralization potencies of mAbs were evaluated in a luciferase assay system. **b** ADE of SARS-CoV-2 pseudoviral infection of nonpermissive Raji and Daudi cells mediated by MW01, MW05 and MW07 were evaluated in a luciferase assay system. SARS-CoV-2 pseudovirus were incubated with 2-fold serially diluted mAbs. The mixtures were then added into Raji or Daudi cells. After 24 h incubation, luciferase activity was measured. **c** Comparison of ADE activity mediated by wild-type and LALA mutated human IgG1 mAbs on Raji cells. **d** ADE of SARS-CoV-2 pseudoviral infection on Raji cells mediated by MW05. Raji cells were pre-treated with anti-FcγRIIA, anti-FcγRIIB or control Fab before inoculation with SARS-CoV-2 pseudovirus. **e** ADE activity of wild-type and Fc region mutated human IgG1 mAbs on Raji cells. “Mu” means G237D, P238D, P271G and A330R combined mutation on human IgG1 mAb, which increases the binding affinity to human FcγRIIB. **f** ADE of SARS-CoV-2 infection mediated by wild type MW05 on monocytes and macrophages. Macrophages were derived from THP-1 and SC monocytes. **g** ADE of SARS-CoV-2 pseudoviral infection mediated by wild type MW05 on Raji cells transiently expressing FcγRIA and FcγRIIA. Asterisks indicate statistically significant differences. (Student’s *t*-test, *P* < 0.05). **h** ADE of SARS-CoV-2 pseudoviral infection on human primary B cells mediated by MW05. Primary B cells were negatively isolated from PBMCs of healthy human donors.

Out of a panel of fully human SARS-CoV-2 RBD binding mAbs, three mAbs, MW01, MW05 and MW07, with high neutralization potency against SARS-CoV-2, were selected as lead mAbs. All three mAbs could inhibit SARS-CoV-2 pseudoviral transduction into Huh7 and Vero cells (Fig. 1a). MW05 and MW07 showed similar neutralization activity, which was stronger than that of MW01 in both tested cells (Fig. 1a). As we reported previously, FcγRIIB, but not FcγRIIA or FcγRIA, was involved in the ADE of SARS-CoV-2 infection on B cells mediated by MW05^22^. FcγRIIB expressing cells Raji and Daudi were used to evaluate the ADE activity of these three SARS-CoV-2 neutralizing mAbs. ADE activity was observed for MW01 and MW05, but not MW07 in both tested cells (Fig. 1b). Daudi cells express not only FcγRIIB, but also FcγRIIA, which may compete for the binding with Fc region of mAbs (Supplementary Fig. 1). As expected, MW01 and MW05 showed a weaker enhancement of SARS-CoV-2 infection on Daudi cells compared with Raji cells (Fig. 1b). ADE activity of MW01 and MW05 was completely abolished by introducing LALA mutation to the Fc portion to disrupt their interaction with FcγRIIB (Fig. 1c and Supplementary Fig. 2a). Blocking the interaction of FcγRIIB/Fc with anti-FcγRIIB specific mAb abrogated ADE of SAR-CoV-2 pseudoviral infection on Raji cells mediated by MW05 (Fig. 1d). We also generated G237D, P238D, P271G and A330R combined mutated mAbs to enhance the binding affinity to FcγRIIB.^23^ Compared with their parental mAbs, FcγRIIB binding ability enhanced mAbs MW01/Mu and MW05/Mu maintained the SARS-CoV-2 neutralization potency, but increased ADE activity (Fig. 1e, Supplementary Figs. 2b and 3).

A hyper-inflammatory response induced by macrophages occurs on patients with severe COVID-19, which subsequently breaks pulmonary endothelial barrier integrity and induces microvascular thrombosis^24^. To investigate whether MW05 induces ADE of SARS-CoV-2 infection on monocytes and macrophages, THP-1 and SC cells were used to differentiate into macrophages in vitro. No ADE activity was observed for MW05 in tested monocytes and macrophages (Fig. 1f). We next checked the expression profiles of FcγRs on these monocytes and macrophages. Much lower FcγRIIB expression was detected for SC, THP-1 and their differentiated macrophages compared with the expression level of high-affinity Fc receptor FcγRIA (Supplementary Fig. 4). To investigate whether other FcγRs expressed on Raji cells could inhibit the ADE activity mediated by MW05, FcγRIA and FcγRIIA were transiently expressed on Raji cells. Decreased ADE activity was observed for MW05 on FcγRIA or FcγRIIA expressing Raji cells compared with wild-type cells (Fig. 1g). Together, FcγRIIB contributes to the ADE activity of SARS-CoV-2 neutralizing mAb MW01 and MW05; the expression of other FcγRs, such as FcγRIA and FcγRIIA may inhibit ADE activity by competing against FcγRIIB for the binding with Fc region of mAbs.

To further prove whether ADE occurs in human primary B cells or not, we performed ADE experiments using primary B cells negatively isolated from PBMCs of healthy human donors. As shown in Fig. 1h, weak ADE signal mediated by MW05 was observed in primary B cells using SARS-CoV-2 pseudovirus, which indicates that ADE of SARS-CoV-2 infection may occur in human.

### Structure of SARS-CoV-2 RBD complex with MW01 or MW05

**Fig. 2:**
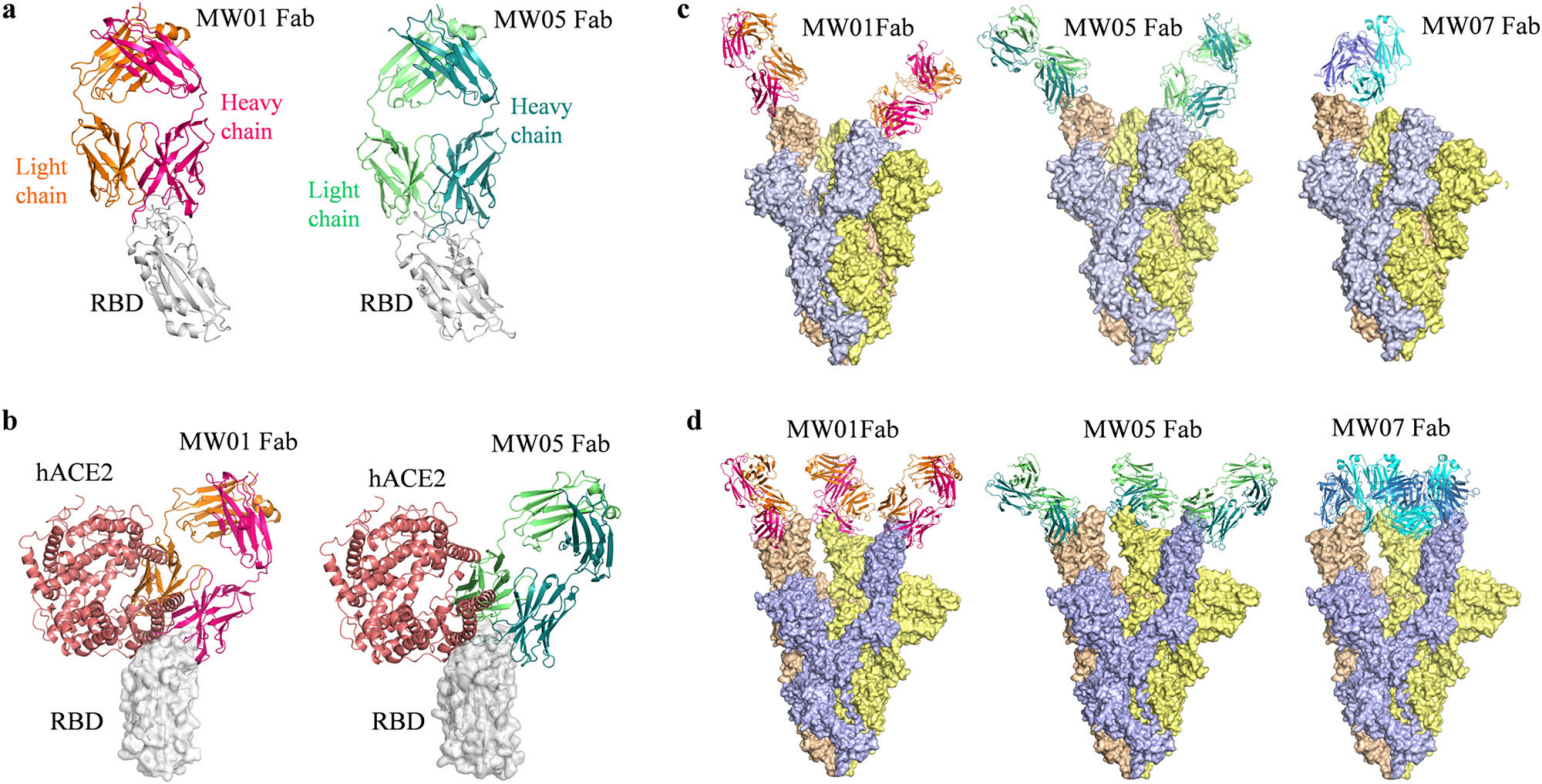
Crystal structure of MW01 and MW05 Fab in complex with SARS-CoV-2 RBD. **a** The overall structure of MW01 and MW05 Fab in complex with SARS-CoV-2 RBD. The SARS-CoV-2 RBD is colored in gray. Heavy chain and light chain of MW01 are shown as hot pink and orange cartoons. Heavy chain and light chain of MW05 are shown as dark green and light green cartoons. **b** Superimposition of MW01/RBD or MW05/RBD with hACE2/RBD. The SARS-CoV-2 RBD is colored in gray surface. Heavy chain and light chain of MW01 and MW05 are presented in cartoon with the same colors in Fig. 2a. hACE2 is colored in salmon. **c** Superimposition of MW01, MW05, and MW07 Fab to partially open SARS-CoV-2 S trimer (PDB code: 6VYB) with 1 “up” RBD and 2 “down” RBDs. For MW01 and MW05, one Fab is superposed to the “up” RBD, and one Fab is superposed to one of the “down” RBDs. Superimposition of Fab to the other “down” RBD will introduce clashes. MW07 Fab can only be superposed to the “open” RBD with no clashes. The heavy chain and light chain of MW01 and MW05 are shown with same colors as Fig. 2a. The heavy chain and light chain of MW07 are colored in blue and light cyan, respectively. The three chains of SARS-CoV-2 S trimer are shown as wheat, gray and yellow surface. **d** Superimposition of MW01, MW05, and MW07 to SARS-CoV-2 S trimer with 3 “up” RBDs (PDB code 7A98). For all these three mAbs, three Fabs are superposed to three “up” RBDs in SARS-CoV-2 trimer. MW01, MW05, MW07 and S trimer as shown with the same colors as Fig. 2c.

To understand the underlying mechanisms of ADE of SARS-CoV-2 infection on FcγRIIB-expressing immune cells mediated by MW01 and MW05, we solved the crystal structures of MW01 and MW05 Fab in complex with SARS-CoV-2 RBD at a resolution of 2.4 Å and 3.2 Å, respectively (Fig. 2a and Table 1). In the complex structure of MW01/RBD, the variable domain of MW01 was bound to the SARS-CoV-2 RBD mainly through its external subdomain (Supplementary Fig. 5). The CDR1, CDR2, and CDR3 of the heavy chain all contributed to RBD recognition (Supplementary Table. 1). Specifically, the side chain of P104 in HCDR3 contacted the hydrophobic side chains of L455 and F456 in RBD. The side chain of G105 in HCDR3 formed hydrogen bonds with the side chains of K417 and Y453 of RBD. The side-chain carboxyl group of E484 of RBD formed hydrogen bonds with the side chains of R50 and N59 in HCDR2, and its main-chain amino and carbonyl groups formed hydrogen groups with the main chains of N59, Y60, and Q62 in HCDR2. The CDR1, CDR2, and the N-terminus of the light chain were involved in antigen recognition (Supplementary Fig. 5).

**Table 1:** Data collection and structure refinement.

	MW01 Fab/RBD	MW05 Fab/RBD
**Data collection**		
Diffraction source	BL19U1, SSRF	BL19U1, SSRF
Detector	Pilatus 6 M	Pilatus 6 M
Wavelength (Å)	0.9785	0.9785
Space group	C222_1_	P3_2_21
Cell dimensions		
a, b, c (Å)	65.62, 86.83, 293.28	87.00, 87.00, 216.50
α, β, γ (°)	90, 90, 90	90, 90, 120
Resolution (Å)	50.00-2.40 (2.49-2.40)*	50.00-3.20 (3.31-3.20)
R_merge_	0.149 (1.110)	0.118 (1.709)
I/σI	16.0 (2.2)	30.9 (2.2)
CC_1/2_	0.994 (0.656)	0.995 (0.773)
Completeness (%)	99.7 (99.7)	100.0 (100.0)
Redundancy	10.6 (10.9)	18.9 (19.9)
Total reflections	354,424	309,722
Unique reflections	33,331	16,375
**Refinement**		
Resolution (Å)	36.68-2.40	36.08-3.20
R_work_/R_free_	0.183/0.230	0.218/0.265
No. of reflections used	33,239	16,241
No. of atoms		
Protein	4877	4786
Ligands	27	14
Water	317	
Average B-factor (Å^2^)		
Protein	47.1	92.3
Ligands	78.5	105.9
Water	45.0	
R.m.s. deviations		
Bond lengths (Å)	0.004	0.004
Bond angles (°)	0.676	0.659
Ramachandran plot		
Favored (%)	95.7	92.7
Allowed (%)	4.3	7.3
Outliers (%)	0	0

^*^Values in parentheses are for the highest-resolution shell.

The comparison of MW01/RBD structure with the hACE2/RBD complex structure^25^ showed that the recognition epitopes of MW01 highly overlapped with the binding surface of hACE2 (Fig. 2b and Supplementary Fig. 6), which indicated that MW01 competed for hACE2 binding. The superimposition of the MW01/RBD structure with the S trimer structures in “closed” or “open” conformations^26,27^ revealed that MW01 could bind to both “down” and “up” RBDs (Fig. 2c). Furthermore, three RBDs in the “closed” conformation could simultaneously bind to three MW01 Fabs without introducing any clashes (Supplementary Fig. 7). For S trimer in the most common “open” conformation^26–31^, in which two RBDs are “down” and one RBD is “up”, the “up” RBD and one “down” RBD could bind two MW01 Fabs without any clashes (Fig. 2c). MW01 Fab binding to another “down” RBD would be blocked by the “up” RBD. For S trimer with 3 “up” RBDs, which was observed in hACE2/S trimer structure^32^ and a Fab/S trimer complex structure^33^, three “up” RBDs could bind three MW01 Fabs (Fig. 2d and Supplementary Fig. 7).

As shown in the complex structure of MW05/RBD, the epitope of MW05 was similar to that of MW01, and the recognition was also dominantly attributed to the heavy chain (Fig. 2a). SARS-CoV-2 RBD recognition involved the CDR1, CDR2, CDR3, and the N-terminus of the heavy chain as well as the CDR2 and CDR3 of the light chain (Supplementary Fig. 8 and Supplementary Table 1). Although the epitopes and binding regions of MW05 and MW01 are similar, the details of the interaction are different (Supplementary Fig. 8). In particular, the HCDR3 of MW05, which was significantly longer than most antibodies and rich in glycine and serine, formed several hydrogen bonds with RBD through their main chains and side chains. The side chain of E484 of RBD, which formed numbers of hydrogen bonds with MW01, also formed several hydrogen bonds with the side-chain or main-chain groups of R50 and N59 in HCDR2 and S108 in HCDR3 (Supplementary Fig. 8).

The structural comparison with the hACE2/RBD structure showed that MW05 binding surface also highly overlapped with the hACE2 binding surface, which indicated that the binding of MW05 to RBD could block the binding of hACE2 (Fig. 2b and Supplementary Fig. 6). Superimposition of MW05/RBD with the S trimer structures showed that the S trimer binding property of MW05 was similar to that of MW01. MW05 could also bind to both “down” and “up” RBDs as MW01 did (Fig. 2c and Supplementary Fig. 9). A “closed” S trimer could bind three MW05 Fabs, an “open” S trimer (1 “up” RBD) could bind to two MW05 Fabs, and a 3 “up” S trimer could bind to three MW05 Fabs (Fig. 2d and Supplementary Fig. 9).

As the complex structure of MW07/RBD (PDB code: 7DK2) have previously been determined, we superposed MW07/RBD to SARS-CoV-2 S trimer with different conformations for side-by-side comparison with the structures of MW01 and MW05. Notably, the S trimer binding property of MW07 which has no ADE activity, is totally different from MW01 and MW05, which could enhance SARS-CoV-2 infection. MW07 only binds to the “up” RBD, but not “down” RBD in both “closed” and “open” S trimers (Fig. 2c and Supplementary Fig. 10). One MW07 Fab binding to any one of the “down” RBDs in the “closed” or “open” S trimers could induce clashes (Fig. 2c and Supplementary Fig. 10). While, in the 3 “up” S trimer, MW07 Fab could bind to all 3 “up” RBDs (Fig. 2d and Supplementary Fig. 10). These results suggest that the epitopes and binding models involve in the ADE activity of SARS-CoV-2 RBD-targeting mAbs.

### Bivalent interaction of MW01 or MW05 with SARS-CoV-2 S trimer

**Fig. 3:**
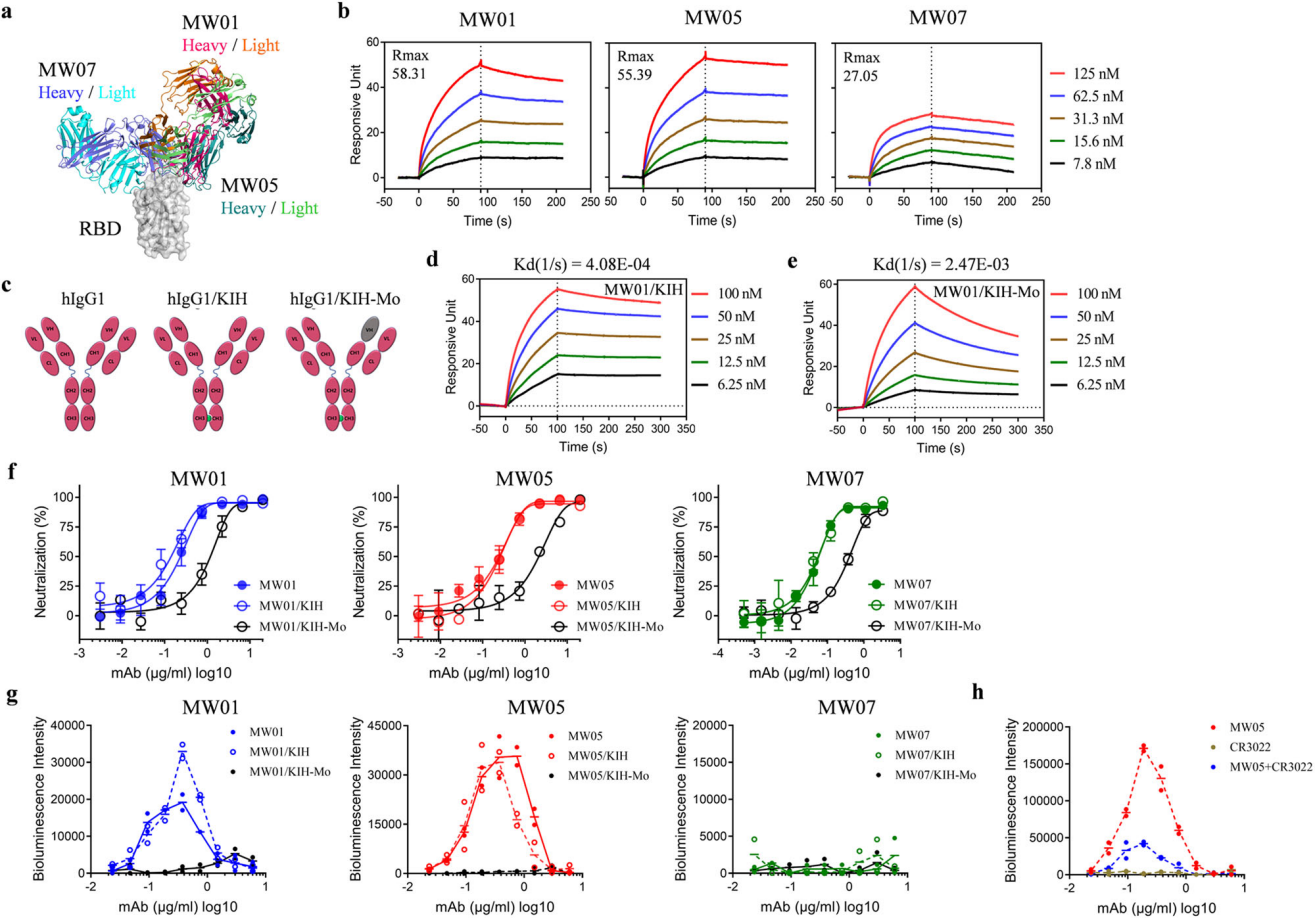
ADE of SARS-CoV-2 infection requires bivalent interaction of mAb with SARS-CoV-2 S trimer. **a** Superimposition of MW01/RBD and MW05/RBD with MW07/RBD. The SARS-CoV-2 RBD is colored in gray surface. Heavy chain and light chain of MW01, MW05, and MW07 are presented in the cartoon with the same colors in Fig. 2. **b** Compare the association signals of MW01, MW05, and MW07 to SARS-CoV-2 S trimer performed by BIAcore T200 system. Recombinant SARS-CoV-2 S trimer protein was captured on the CM5 chip via anti-his antibody at about 200 response units. 2-fold serially diluted MW01, MW05, and MW07 antibody starting from 125 nM were then flowed over the chip surface. **c** Schematic illustration of the wild-type mAb (hIgG1), knob-in-hole format bivalent mAb (hIgG1/KIH) and monovalent mAb (hIgG1/KIH-Mo). hIgG1/KIH-Mo was generated by substitution of hole chain VH domain with an irrelevant antibody. The irrelevant VH domain is colored with gray. **d, e** Measurement of the dissociation signals of MW01/KIH and MW01/KIH-Mo. NTA biosensor chip was used to capture SARS-CoV-2 S trimer on BIAcore S200 system. 2-fold serially diluted MW01/KIH and MW01/KIH-Mo starting from 100 nM were then flowed over the chip surface. **f** Compare the SARS-CoV-2 neutralization potencies of wild-type, KIH format bivalent, and monovalent antibodies using SARS-CoV-2 pseudovirus system. **g** Compare the ADE of SARS-CoV-2 infection on Raji cells mediated by wild-type, KIH format bivalent, and monovalent antibodies. **h** Compare the ADE of SARS-CoV-2 infection on Raji cells mediated by MW05, CR3022, and MW05/CR3022 mixture.

MW01 and MW05, which showed ADE of SARS-CoV-2 infection could bind to both “up” and “down” RBDs in S trimer. While, MW07, a binding competitor of MW01 and MW05, which has no ADE activity, only binds to “up” RBD in S trimer (Fig. 3a). To investigate the different binding models of MW01, MW05, and MW07, we measured the association signals of these three mAbs in BIAcore system by coating S trimer recombinant protein on the chips. Interestingly, the association signal Rmax (RU) of MW01 and MW05 is about 2-fold higher than that of MW07 (Fig. 3b). This data implies that each S trimer may bind to two MW01 or MW05, but only one MW07. To further determine whether ADE of SARS-CoV-2 infection requires bivalent interaction of mAbs with SARS-CoV-2 S trimer, we generated bivalent and monovalent mAbs using knob-in-hole (KIH) strategy^34^ to maintain intact Fc and Fab binding activity (Fig. 3c). Avidities and dissociation signals of bivalent and monovalent mAbs to SARS-CoV-2 S trimer were measured by BIAcore S200 system. Compared with their parental bivalent mAbs, the dissociation signals kd (1/s) of monovalent MW01 (MW01/KIH-Mo) and MW05 (MW05/KIH-Mo) dropped 6.1 and 1.8 folds, respectively (Fig. 3d, e and Supplementary Fig. 11). While less than 1-fold of dissociation signal change was observed for bivalent and monovalent MW07 (Supplementary Fig. 11). These results indicate that MW01 and MW05 exhibit bivalent interaction with SARS-CoV-2 S trimer, and MW07 has monovalent binding with S trimer.

We then tested the SARS-CoV-2 neutralization potencies of these monovalent and bivalent mAbs. As expected, all KIH format bivalent mAbs MW01/KIH, MW05/KIH and MW07/KIH showed similar levels of neutralization potencies compared with their parental wild-type hIgG1 format mAbs (Fig. 3f). The neutralization potency of all monovalent mAbs MW01/KIH-Mo, MW05/KIH-Mo, and MW07/KIH-Mo decreased compared with their parental bivalent mAbs (Fig. 3f). Next, we assessed the ADE of SARS-CoV-2 infection on Raji cells mediated by these bivalent and monovalent mAbs. As expected, the KIH format bivalent mAbs MW01/KIH and MW05/KIH showed similar level of ADE activity compared with their parental hIgG1 format mAbs (Fig. 3g). Notably, monovalent mAbs MW01/KIH-Mo and MW05/KIH-Mo completely abolished the ADE activity, demonstrating that ADE of SARS-CoV-2 infection on Raji cells mediated by MW01 and MW05 requires bivalent interaction of mAbs with SARS-CoV-2 S trimer (Fig. 3g).

In the physiological condition of SARS-CoV-2 vaccination, polyclonal antibodies targeting multiple epitopes would be induced. To test whether ADE activity of MW05 could be inhibited in the presence of antibodies targeting other epitopes, we compared the ADE of SARS-CoV-2 infection on Raji cells mediated by MW05 and MW05 together with CR3022, which recognizes a epitope different from MW05^35^. The result showed that CR3022 decreased the ADE activity of MW05 (Fig. 3h). Sera from different COVID-19 patients could also inhibit the ADE signal mediated by MW05 (Supplementary Fig. 12), indicating that the ADE activity of MW05 or MW05-like antibodies could be inhibited by other non-ADE antibodies.

### ADE mediated SARS-CoV-2 pseudoviral entry

**Fig. 4:**
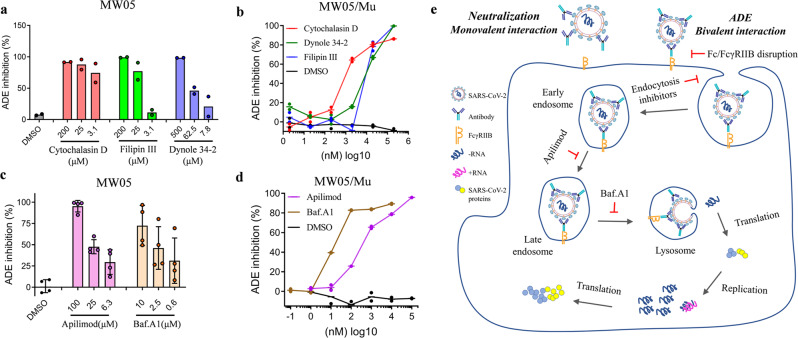
FcγRIIB-mediated uptake and transport of SARS-CoV-2/mAb complex. **a, b** Dose-dependent inhibition of ADE activity of MW05 and MW05/Mu on Raji cells by 3 compounds. The initial concentrations were 200 μM for Cytochalasin D and Filipin III; 500 μM for Dynole 34-2. DMSO was used as solvent control. **c**, **d** Dose-dependent Inhibition of ADE activity of MW05 and MW05/Mu by two endosome transport and acidification inhibitors Apilimod and Baf.A1. The initial concentrations were 100 μM for Apilimod and 10 μM for Baf.A1. DMSO was used as solvent control. Error bars show standard deviation from 4 biological replicates. **e** Schematic diagram showing one of the ADE mechanisms of SARS-CoV-2. For FcγRIIB expressing cells, mAbs enhance SARS-CoV-2 infection via FcγRIIB-mediated uptake of virus and mAb complex with bivalent interaction, resulting in the infection of SARS-CoV-2 on non-permissible Raji and Daudi cells. After entry into the cells, the complex will go through the early endosome, late endosome to lysosome, where the viral capsid is removed, and the viral genome is released. The targets for inhibition of ADE of SARS-CoV-2 are labeled.

To explore how SARS-CoV-2/mAb complex enter Raji cells mediated by FcγRIIB, 3 inhibitors targeting different uptake processes were evaluated. All these 3 inhibitors, the macropinocytosis inhibitor Cytochalasin D, the clathrin-dependent endocytosis inhibitor Dynole 34-2 and caveolin-dependent endocytosis inhibitor Filipin III showed dose-dependent inhibition of ADE activity mediated by MW05 (Fig. 4a). This pattern was also observed for MW05/Mu, a previously generated ADE activity increased mAb (Fig. 4b). These results suggest that both macropinocytosis and endocytosis are involved in ADE of SARS-CoV-2 infection on Raji cells.

To determine the intracellular trafficking patterns after FcγRIIB-mediated SARS-CoV-2/mAb complex entry, the phosphoinositide 5-kinase (PIKfyve) inhibitor Apilimod and the lysosome acidification inhibitor bafilomycin A1 (Baf.A1) were used to inhibit ADE activity of MW05 and MW05/Mu. Apilimod and Baf.A1 significantly diminished the ADE of SARS-CoV-2 infection by MW05 and MW05/Mu (Fig. 4c, d). No difference was observed for FcγRIIB phosphorylation induced by the complex of SARS-CoV-2 pseudovirus together with MW01, MW05 or MW07 (Supplementary Fig. 13). This data implies that, after FcγRIIB-mediated uptake, SARS-CoV-2/mAb complex transports to lysosome followed by membrane fusion and viral genome release (Fig. 4e).

## Discussion

ADE has been observed in SARS, MERS, and other human respiratory viral infections including RSV and measles, which suggests a real risk of ADE for SARS-CoV-2 vaccines and antibody-based interventions. It is important to investigate the potential safety risk of ADE to evaluate vaccines and neutralizing antibody interventions, and research on the mechanism of ADE is helpful to support treatment strategy optimization. Here, we reported a novel mechanism of ADE of SARS-CoV-2 pseudoviral infection on Raji or Daudi cells in vitro (Fig. 4e).

ADE can be broadly categorized into two different types in viral infections, enhanced infections and enhanced immune activation. In the manner of enhanced infections, higher infections are mediated by Fc-FcγR interaction^36^. ADE via FcγRIIA-mediated endocytosis into phagocytic cells can be observed in vitro and has been extensively studied for macrophage-tropic viruses, including dengue virus^37^ in humans and FIPV in cats^38^. Here, our study revealed that the ADE of SARS-CoV-2 pseudovirus mediated by neutralizing mAbs, MW01 and MW05 on Raji and Daudi B cells depended on FcγRIIB instead of FcγRIIA. MW01 and MW05 exhibited significant ADE effects on FcγRIIB single Fcγ receptor-expressing Raji cell. For FcγRIIA and FcγRIIB co-expressing Daudi and K562 cells, the ADE responsive value diminished or eliminated, respectively. These results indicate a positive correlation between FcγRIIB and ADE. ADE via FcγRIIB-mediated endocytosis into FcγRIIB-expressing B cell has also been reported in the research of SARS-CoV^39,40^. Our results provide evidence that the mechanisms of ADE vary among different kinds of virus, and the mechanism of SARS-CoV-2 is similar with that of SARS-CoV. Thus, blocking the Fc-FcγR interaction through Fc mutation can be an effective strategy to eliminate the potential ADE in vivo. Even though we demonstrate that loss of bivalent binding leads to loss of ADE mediated by several SARS-CoV-2 neutralizing mAbs developed by our group and other teams, the underlying mechanism of how the monovalent binding loss of ADE is still elusive. Perhaps bivalent interaction could induce more stronger cross-linking and formation of immune complex. Another factor may be the conformation change of S-trimer induced by the bivalent interaction. Further studies are needed to uncover the link between the bivalent interaction of mAb and ADE.

An epitope-ADE relationship has also been reported for MERS-CoV^41^. In this study, similar phenomenon was observed. MW01 and MW05, which recognize the same epitope bin, show significant ADE activities, while CR3022, which targets a different epitope, has no ADE activity. Meanwhile, both CR3022 and serum from COVID-19 convalescent patients can inhibit the ADE effects of MW05, indicating that among the polyclonal antibodies induced by SARS-CoV-2 infection, only the minority of the antibodies in the sera has the potential to cause ADE. What’s more, we found that monovalent or bivalent mAb binding with the same epitope shows different ADE effects. These results suggest that, besides epitopes, binding models also involve in the ADE activity of SARS-CoV-2 RBD-targeting mAbs. These results might shed light for optimizing the antibody-based therapeutics.

Overall, our results provide a novel insight of the mechanism of antibody-enhanced SARS-CoV-2 pseudoviral infection on FcγRIIB-expressing B cells. This study is informative to better understand the process of ADE of SARS-CoV-2 and provide potential strategies to mitigate ADE activity induced by antibody-based therapeutics in development. The limitation of this study is that the potential ADE mechanism was identified and confirmed using SARS-CoV-2 pseudovirus-based experiments. As we could not get access to P3 Laboratory platform at this moment, whether this ADE mechanism could be further confirmed using SARS-CoV-2 authentic virus is not quite sure. In future, SARS-CoV-2 authentic virus-based experiments and animal studies are needed to further evaluate this ADE mechanism for SARS-CoV-2 in vitro and in vivo.

## Methods

### Materials

HEK293 (ATCC, CRL-3216) cells, Huh7 (Institute of Basic Medical Sciences CAMS, 3111C0001CCC000679) cells and Vero (ATCC, CCL-81) cells were cultured at 37 °C in Dulbecco’s Modified Eagle medium (DMEM, Hyclone SH30243.01) supplemented with 10% fetal bovine serum (FBS). CHO-K1 (ATCC, CCL-61) cells were cultured at 37 °C in Dynamis Medium (GIBCO A2661501). Raji (ATCC, CCL-86) cells, THP-1 (ATCC, TIB-202) cells, K562 (ATCC, CCL-243) cells, and SC (ATCC, CRL-9855) cells were cultured at 37 °C in RPMI 1640 Medium (BasalMedia L220KJ) with 10% FBS. SARS-CoV-2 pseudovirus was prepared and provided by the Institute for Biological Product Control, National Institutes for Food and Drug Control (NIFDC)^42^.

### Neutralization assay

For pseudovirus neutralization assay, 100 µL of mAbs at different concentrations were mixed with 50 µL supernatant containing 1000 TCID_50_ pseudovirus. The mixture was then incubated for 60 min at 37 °C, supplied with 5% CO_2_. 100 µL of Huh 7 cell or Vero cell suspension (2 × 10^5^ cells/mL) was then added to the mixtures of pseudoviruses and mAbs for an additional 24 h incubation at 37 °C. Then, 150 μL of supernatant was removed, and 100 μL luciferase detecting regents (PerkinElmer 6066769) was added to each well. After 2 min of incubation, each well was mixed 6-8 times by pipetting, and 150 μL of the mixture was transferred to a new microplate. Luciferase activity was measured using a microplate luminometer (SpectraMax L MolecularDevices). The neutralization titer was calculated using GraphPad Prism 7.0.

### Antibody-dependent enhancement (ADE) assay

The ADE assays were performed using Raji, THP-1, K562, SC and Daudi cell lines. 25 µL of serially diluted mAbs or mAbs combinations were mixed with 25 µL supernatant containing 750 TCID_50_ pseudovirus. The mixture was incubated for 60 min at 37 °C, supplied with 5% CO_2_. 50 µL cells at the density of 2 × 10^6^ cells/mL were added to the mixtures of pseudoviruses and mAbs for an additional 24 h incubation. Then, the same volume of luciferase detecting regents (Promega G7940) was added to each well. After 2 min of incubation, the luciferase activity was measured using a microplate luminometer (SpectraMax i3x MolecularDevices).

### ADE inhibition assay

The ADE inhibition assay were performed using Raji cells. Diluted mAbs (MW05: 750 ng/mL and MW05/Mu: 200 ng/mL) were mixed with pseudovirus. The mixture was incubated for 60 min at 37 °C, supplied with 5% CO_2_. 25 µL of serially diluted inhibitors of micropinocytosis and endocytosis. (Dynole, Abcam ab120346; methyl-β-cyclodextrin, Sigma C4555-1G, Thioridazine, Sigma T9025-5G; Apilimod, MCE HY-14644; Cytochalasin D, MCE HY6682; Fillipin, CAYMAM CHEMICAM COMPANY 70400; Baf.A1,Cell Signaling 56545 S) were mixed with 25 µL cells at the density of 4 × 10^6^ cells/mL. Then add the mixtures of pseudoviruses and mAbs to the cell plate for an additional 24 h incubation. Then, the same volume of luciferase detecting regents (Promega G7940) was added to each well. After 2 min of incubation, the luciferase activity was measured using a microplate luminometer (SpectraMax i3x MolecularDevices). For evaluation whether FcγRI expression on B cells could impact the ADE mediated by MW05 or not, the construct FcγRI-P2A-gamma chain driven by SFFV promoter was generated. Raji cells were used for ADE measurement after transfected by FcγRI-P2A-gamma chain construct with electroporation strategy.

### Generation of mutated mAbs to increase the binding to FcγRIIB

For preparation of mutated mAbs to increase the binding to FcγRIIB, heavy chain and light chain plasmids were transiently co-transfected into HEK293 cells using 293fectinTM Transfection Reagent (Life Technologies 12347019) followed by purification with Mabselect TM (Cytiva 17-5199-02). The heavy chain of these mutated mAbs were introduced the G237D, P238D, P271G, and A330R combined mutation to their parental hIgG1 heavy chain vectors.

### Monocytes differentiation

For preparing the macrophages, 60 ng/ml P P8139MA (Sigma-Aldrich P8139) was used to induce the differentiation of SC and THP-1 cells. Cells at a density of 5 × 10^5^/mL were seeded at T75 flasks (Corning 430641) and cultured for 6 h at 37 °C, supplied with 5% CO_2_. Then, 100 ng/ml LPS (Sigma-Aldrich L6529) were added to induce further differentiation of SC and THP-1 cells for additional 18 h. After that, media was changed, and cells were cultured in the media with 60 ng/ml PMA plus 100 ng/ml LPS for another 48 h. Macrophages were then collected for FcγR detection and ADE assays.

### Flow cytometry assay

The FcγR expression profiles of Raji, Daudi, THP-1, THP-1-derived macrophage, SC, SC-derived macrophage, and K562 were determined by FACS. Cells were collected and washed twice with 1×PBS, then blocked with Fc receptor blocking buffer for 20 min at RT. 10 μL FITC conjugated anti-FcγRIA antibody (Sino Biological 10256-R401-F), FITC conjugated anti-FcγRIIA antibody (Sino Biological 10374-MM01-F), FITC conjugated anti-FcγRIIIA antibody (Sino Biological 10389-MM41-F) or 1 μg anti-FcγRIIB antibody(Biolegend 398302) labeled with FITC using labeling Kit (Sigma-Aldrich MX488AS100) into cells (1 × 10^6^ cells/sample in 100 μL) and incubated for 60 min at 2–6°C and analyzed using flow cytometry (CytoFLEX, Beckman Coulter).

### Surface plasmon resonance (SPR)

All SPR measurements were performed at 25 °C. For checking the association signals of MW01, MW05, and MW07, BIAcore T200 system with CM5 biosensor chips (Cytiva BR100012) wase used. A buffer consisting of 150 mM NaCl, 10 mM HEPES, 3 mM EDTA, pH 7.4 and 0.005% (v/v) Tween-20 was used as running buffer. The blank channel of the chip served as the negative control. Recombinant SARS-CoV-2 S trimer protein (ACRO Biosystems SPN-C52H9) was captured on the chip via His Capture Kit (Cytiva 28995056) at about 200 response units. Gradient concentrations of MW01, MW05, and MW07 antibody (from 125 nM to 7.8125 nM with 2-fold dilution) were then flowed over the chip surface. After each cycle, the sensor was regenerated with Gly-HCl (pH 1.5). For measuring the affinity of bivalent and monovalent antibodies for MW01, MW05, and MW07, BIAcore S200 system with NTA biosensor chips (Cytiva 28994951). A buffer consisting of 150 mM NaCl, 10 mM HEPES, 3 mM EDTA, pH 7.4 and 0.05% (v/v) Surfactant P20 pH 7.4 was used as running buffer. All proteins were normalized concentration by using this buffer in advance. The blank channel of the chip served as the negative control. Recombinant SARS-CoV-2 S trimer protein (ACRO Biosystems SPN-C52H9) was captured on the chip at 75 response units. Gradient concentrations of bivalent and monovalent antibodies (from 100 nM to 6.25 nM with 2-fold dilution) were then flowed over the chip surface. After each cycle, the sensor was regenerated with 0.35 M EDTA. The affinity was calculated using a 1:1 (Rmax Global fit) binding fit model with BIAcore evaluation software.

### Generation of Fab/RBD complex

SARS-CoV-2 RBD recombinant protein was produced as previously described^43^. For the preparation of MW01 Fab/RBD complex, SARS-CoV-2 RBD recombinant protein tagged with a N-terminal 6×His tag together with MW01 Fab were mixed at a molar ratio of 1:1 in a 20 mM Tris, 150 mM NaCl buffer. After incubation at room temperature for 2 h, the mixture was concentrated by Ultrafiltration concentrator (Sartorius Stedim VS2002) to a concentration of 20 mg/mL in a volume of 0.3 mL. SEC-HPLC and SDS-PAGE were used to check the size and purity. For the preparation of MW05 Fab/RBD complex, SARS-CoV-2 RBD recombinant protein tagged with a N-terminal 6×His tag and MW05 Fab were mixed together at a molar ratio of 1:2.5 in a 20 mM Tris,150 mM NaCl buffer. After incubation at room temperature for 2 h, the mixture was purified by HisTrapTM HP (GE Healthcare 17-5248-01), and then concentrated by Ultrafiltration concentrator (Sartorius Stedim VS2002) to a concentration of 23 mg/mL in a volume of 0.3 mL. SEC-HPLC and SDS-PAGE were used to check the size and purity.

### Crystallization, structure determination, and Spike trimer-binding modeling

The crystallization was performed at 16 °C using the sitting-drop vapor-diffusion method. The crystals of the SARS-CoV-2 RBD/MW01 Fab complex were obtained in a condition of 0.1 M sodium citrate, pH 5.6, 18% (w/v) polyethylene glycol (PEG) 4000, 14% (v/v) 2-propanol, and 0.01 M barium chloride. The crystals of the SARS-CoV-2 RBD/MW05 Fab complex were obtained in 0.2 M sodium citrate tribasic, 21% (w/v) PEG 3350, and 0.02 M urea. The crystals were soaked in reservoir solutions with additional 10% glycerol for several seconds and flash-frozen in liquid nitrogen before diffraction data collection.

All the X-ray diffraction data were collected at beamline BL19U1^44^ of the Shanghai Synchrotron Radiation Facility (SSRF) with a wavelength of 0.9785 Å, using a Pilatus 6 M detector, and processed with HKL3000 software^45^. The structures were resolved using molecular replacement method with PHASER^46^ in CCP4 suite^47^, using the SARS-CoV-2 RBD structures in the SARS-CoV-2 RBD/hACE2 complex (PDB code 6LZG)^25^ and the Fab structure in the MW317/PD1 complex (PDB code 6JJP)^48^ as the search models. The final models were rebuilt with COOT^49^ and refined with PHENIX^50^. Detailed statistics for data collection and structure determination are summarized in Table 1.

The models of Fabs bound to “closed”, “1 up”, and “3 up” S trimers were generated by superimposing the RBDs in the RBD/Fab complexes with RBDs in the “closed” S trimer (PDB code 6VXX)^26^, “1 up” S trimer (PDB code 6VYB)^26^, and “3 up” S trimer/hACE2 complex (PDB code 7A98)^32^ structures using CCP4^50^, respectively.

### Generation of KIH format bivalent and monovalent mAbs

For generation of KIH format monovalent MW01 (MW01/KIH-Mo), MW05 (MW05/KIH-Mo) and MW07 (MW07/KIH-Mo), the coding sequences of light chains were cloned into the upstream of the human Kappa light chain constant region in a eukaryotic expression vector pKN019 with restriction digestion. The coding sequences of heavy chains were inserted into pKN009-A plasmid containing a human IgG1 heavy chain constant region sequence with T336Y mutation^34^. Heavy chain sequence from a irrelative antibody was inserted into pKN009-A vector Y407T mutation in the Fc region (pKN009-B). MW01/KIH-Mo, MW05/KIH-Mo and MW07/KIH-Mo were expressed by transient co-transfection of pKN019, pKN009-A(T336Y), and pKN009-B(Y407T) into HEK293 cells using 293fectinTM Transfection Reagent (Life Technologies 12347019). After 4 days, the supernatant was collected by centrifugation followed by purification using Mabselect TM (Cytiva 17-5199-02). This purified protein was buffer exchanged into PBS using a Vivacon 500 concentrator (Sartorius Stedim VS0122). SEC-HPLC and SDS-PAGE were used to check the size and purity of these mAbs.

For the generation of KIH format bivalent MW01 (MW01/KIH), MW05 (MW05/KIH), and MW07 (MW07/KIH), the VH sequences were cloned in the upstream of the human IgG1 heavy chain constant region in two different expression vectors pKN009-A (T336Y) and pKN009-B (Y407T) separately. Light chains were constructed in the process described above. Light chain expression plasmid and two different heavy chain expression plasmids were transiently co-transfected into HEK293 cells to produce KIH format bivalent mAbs. The purification and identification process are the same as the preparation of KIH format bivalent mAbs mentioned above.

### Statistics and reproducibility

Statistical testing was completed using Prism 7. Samples were randomly allocated into experimental groups, and no data were excluded from the analysis. All replicates used for statistical testing represent biological replicates.

### Reporting summary

Further information on research design is available in the Nature Research Reporting Summary linked to this article.

## Supplementary information

Supplementary Information

Description of Additional Supplementary Files

Supplementary Data 1

Reporting Summary

## Data Availability

Source data for the graphs and charts in the main figures is provided as Supplementary Data 1 and any remaining information can be obtained from the corresponding author upon reasonable request. The atomic coordinate for the complex of SARS-CoV-2 RBD/MW01, RBD/MW05 and RBD/MW07 have been deposited in the Protein Data Bank (www.rcsb.org). The PDB ID codes for RBD/MW01, RBD/MW05 and RBD/MW07 are 7DKZ, 7DK0, and 7DK2, respectively.

## References

[1] Callaway E, Cyranoski D, Mallapaty S, Stoye E, Tollefson J (2020). The coronavirus pandemic in five powerful charts. Nature.

[2] Krammer, F. SARS-CoV-2 vaccines in development. *Nature* **585**, 174–177 (2020). 10.1038/s41586-020-2798-3 32967006

[3] Xia, S. et al. Safety and immunogenicity of an inactivated SARS-CoV-2 vaccine, BBIBP-CorV: a randomised, double-blind, placebo-controlled, phase 1/2 trial. *Lancet Infect. Dis.* **22**, 196–208, (2020). 10.1016/S1473-3099(20)30831-8 PMC7561304 33069281

[4] Keech, C. et al. Phase 1-2 Trial of a SARS-CoV-2 Recombinant Spike Protein Nanoparticle Vaccine. *N. Engl. J. Med.* **383**, 2320–2332 (2020). 10.1056/NEJMoa2026920 PMC7494251 32877576

[5] Jackson, L. A. et al. An mRNA Vaccine against SARS-CoV-2 - Preliminary Report. *N. Engl. J. Med.* **383**, 1920–1931 (2020). 10.1056/NEJMoa2022483 PMC7377258 32663912

[6] van Doremalen N (2020). ChAdOx1 nCoV-19 vaccine prevents SARS-CoV-2 pneumonia in rhesus macaques. Nature.

[7] Shi, R. et al. A human neutralizing antibody targets the receptor binding site of SARS-CoV-2. *Nature* **584**, 120–124 (2020). 10.1038/s41586-020-2381-y 32454512

[8] Baum, A. et al. REGN-COV2 antibody cocktail prevents and treats SARS-CoV-2 infection in rhesus macaques and hamsters. *Nature.* **370**, 1110–1115 (2020). 10.1126/science.abe2402 PMC7857396 33037066

[9] Liu, L. et al. Potent neutralizing antibodies against multiple epitopes on SARS-CoV-2 spike. *Nature* **584**, 450-456 (2020). 10.1038/s41586-020-2571-7 32698192

[10] S. Du et al. Structurally resolved SARS-CoV-2 antibody shows high efficacy in severely infected hamsters and provides a potent cocktail pairing strategy. *Cell*. **183**, 1013–1023 (2020). 10.1016/j.cell.2020.09.035 PMC7489885 32970990

[11] Focosi D, Franchini M (2021). COVID-19 convalescent plasma therapy: hit fast, hit hard!. Vox Sang..

[12] Corti D, Purcell LA, Snell G, Veesler D (2021). Tackling COVID-19 with neutralizing monoclonal antibodies. Cell.

[13] Graham, B. S. Vaccines against respiratory syncytial virus: the time has finally come. *Vaccine*. **34**, 3535–3541 (2016). 10.1016/j.vaccine.2016.04.083 PMC4912855 27182820

[14] Stettler, K. et al. Specificity, cross-reactivity, and function of antibodies elicited by Zika virus infection. *Science* **353**, 823–826 (2016). 10.1126/science.aaf8505 27417494

[15] Wang, T. T. et al. IgG antibodies to dengue enhanced for FcγRIIIA binding determine disease severity. *Science* **355**, 395–398 (2017). 10.1126/science.aai8128 PMC5557095 28126818

[16] Liu, L. et al. Anti–spike IgG causes severe acute lung injury by skewing macrophage responses during acute SARS-CoV infection. *JCI Insight.* **4**, e123158 (2019). 10.1172/jci.insight.123158 PMC6478436 30830861

[17] Agrawal, A. S. et al. Immunization with inactivated Middle East Respiratory Syndrome coronavirus vaccine leads to lung immunopathology on challenge with live virus. *Hum. Vaccin. Immunother.* **12**, 2351–2356 (2016). 10.1080/21645515.2016.1177688 PMC5027702 27269431

[18] Lee, W. S., Wheatley, A. K., Kent, S. J. & DeKosky, B. J. Antibody-dependent enhancement and SARS-CoV-2 vaccines and therapies. *Nat. Microbiol*. **5**, 1185–1191 (2020). 10.1038/s41564-020-00789-5 PMC12103240 32908214

[19] Wan, Y. et al. Molecular mechanism for antibody-dependent enhancement of coronavirus entry. *J. Virol.* **94**, e02015–19 (2020). 10.1128/JVI.02015-19 PMC7022351 31826992

[20] Willey, S. et al. Extensive complement-dependent enhancement of HIV-1 by autologous non-neutralising antibodies at early stages of infection. *Retrovirology* **8**, 16 (2011). 10.1186/1742-4690-8-16 PMC3065417 21401915

[21] Walls, A. C. et al. Unexpected receptor functional mimicry elucidates activation of coronavirus fusion. *Cell* **176**, 1026–1039. e1015 (2019). 10.1016/j.cell.2018.12.028 PMC6751136 30712865

[22] Wang S (2020). Characterization of neutralizing antibody with prophylactic and therapeutic efficacy against SARS-CoV-2 in rhesus monkeys. Nat. Commun..

[23] Dahan, R. et al. Therapeutic activity of agonistic, human anti-CD40 monoclonal antibodies requires selective FcγR engagement. *Cancer Cell* **29**, 820–831 (2016). 10.1016/j.ccell.2016.05.001 PMC4975533 27265505

[24] Hoepel, W. et al. Anti-SARS-CoV-2 IgG from severely ill COVID-19 patients promotes macrophage hyper-inflammatory responses. *bioRxiv* (2020).

[25] Wang Q (2020). Structural and Functional Basis of SARS-CoV-2 Entry by Using Human ACE2. Cell.

[26] Walls AC (2020). Structure, Function, and Antigenicity of the SARS-CoV-2 Spike Glycoprotein. Cell.

[27] Ke, Z. et al. Structures and distributions of SARS-CoV-2 spike proteins on intact virions. *Nature* **588**, 498–502 (2020). 10.1038/s41586-020-2665-2 PMC7116492 32805734

[28] Yurkovetskiy, L. et al. Structural and Functional Analysis of the D614G SARS-CoV-2 Spike Protein Variant. *Cell* **1873**, 739–751 (2020). 10.1016/j.cell.2020.09.032 PMC7492024 32991842

[29] Cai Y (2020). Distinct conformational states of SARS-CoV-2 spike protein. Science.

[30] Henderson R (2020). Controlling the SARS-CoV-2 spike glycoprotein conformation. Nat. Struct. Mol. Biol..

[31] Wrapp D (2020). Cryo-EM structure of the 2019-nCoV spike in the prefusion conformation. Science.

[32] Benton, D. J. et al. Receptor binding and priming of the spike protein of SARS-CoV-2 for membrane fusion. *Nature.* **588**, 327–330 (2020). 10.1038/s41586-020-2772-0 PMC7116727 32942285

[33] Barnes CO (2020). Structures of Human Antibodies Bound to SARS-CoV-2 Spike Reveal Common Epitopes and Recurrent Features of Antibodies. Cell.

[34] Ridgway JB, Presta LG, Carter P (1996). ‘Knobs-into-holes’ engineering of antibody CH3 domains for heavy chain heterodimerization. Protein Eng..

[35] Yuan, M. et al. A highly conserved cryptic epitope in the receptor binding domains of SARS-CoV-2 and SARS-CoV. *Science* **368**, 630–633 (2020). 10.1126/science.abb7269 PMC7164391 32245784

[36] Lee WS, Wheatley AK, Kent SJ, DeKosky BJ (2020). Antibody-dependent enhancement and SARS-CoV-2 vaccines and therapies. Nat. Microbiol.

[37] Halstead SB, O’Rourke EJ (1977). Dengue viruses and mononuclear phagocytes. I. Infection enhancement by non-neutralizing antibody. J. Exp. Med.

[38] Hohdatsu T (1998). Antibody-dependent enhancement of feline infectious peritonitis virus infection in feline alveolar macrophages and human monocyte cell line U937 by serum of cats experimentally or naturally infected with feline coronavirus. J. Vet. Med Sci..

[39] Jaume M (2011). Anti-severe acute respiratory syndrome coronavirus spike antibodies trigger infection of human immune cells via a pH- and cysteine protease-independent FcgammaR pathway. J. Virol..

[40] Kam YW (2007). Antibodies against trimeric S glycoprotein protect hamsters against SARS-CoV challenge despite their capacity to mediate FcgammaRII-dependent entry into B cells in vitro. Vaccine.

[41] Zhou Y (2021). Enhancement versus neutralization by SARS-CoV-2 antibodies from a convalescent donor associates with distinct epitopes on the RBD. Cell Rep..

[42] Nie, J. et al. Establishment and validation of a pseudovirus neutralization assay for SARS-CoV-2. *Emerg. Microbes Infect.* **9**, 680–686 (2020). 10.1080/22221751.2020.1743767 PMC7144318 32207377

[43] Wang, S. et al. An antibody-dependent enhancement (ADE) activity eliminated neutralizing antibody with potent prophylactic and therapeutic efficacy against SARS-CoV-2 in rhesus monkeys. *Nat. Commun*. **11**, 5752 (2020). 10.1038/s41467-020-19568-1 PMC7666115 33188207

[44] Zhang W-Z (2019). Protein complex Crystallogr. beamline (BL19U1) Shanghai Synchrotron Radiat. Facil..

[45] Otwinowski Z, Minor W (1997). Processing of X-ray diffraction data collected in oscillation mode. Methods Enzymol..

[46] McCoy AJ (2007). Phaser crystallographic software. J. Appl. Crystallogr..

[47] Winn MD (2011). Overview of the CCP4 suite and current developments. Acta Crystallogr D. Biol. Crystallogr.

[48] Wang M (2019). Identification of a monoclonal antibody that targets PD-1 in a manner requiring PD-1 Asn58 glycosylation. Commun. Biol..

[49] Emsley P, Cowtan K (2004). Coot: model-building tools for molecular graphics. Acta Crystallogr D. Biol. Crystallogr.

[50] Adams PD (2010). PHENIX: a comprehensive Python-based system for macromolecular structure solution. Acta Crystallogr D. Biol. Crystallogr.

