# Supplementary material for: Antibody-dependent enhancement (ADE) of SARS-CoV-2 pseudoviral infection requires FcγRIIB and virus-antibody complex with bivalent interaction
Source: Commun Biol. 2022 Mar 24;5:262. doi: 10.1038/s42003-022-03207-0 (PMC8948278; doi:10.1038/s42003-022-03207-0)
Supplement: Supplementary file 1 — Supplementary Information [file 42003_2022_3207_MOESM1_ESM.pdf]

Supplementary Information

Title: Antibody-dependent enhancement (ADE) of SARS-CoV-2 pseudoviral infection requires FcγRIIB and virus-antibody complex with bivalent interaction

Authors:
Shuang Wang[1,2,*] Junchao Wang[3,*], Xiaojuan Yu[4,*], Wen Jiang[1,*], Shuo Chen[5,*], Rongjuan Wang[1,2,*], Mingzhu Wang[3], Shasha Jiao[1,2], Yingying Yang[1,2], Wenbo Wang[4], Huilin Chen[1], Ben Chen[1], Chunying Gu[1], Chuang Liu[1], An Wang[1], Min Wang[1], Gang Li[1], Cuicui Guo[1], Datao Liu[1], Jinchao Zhang[1✉], Min Zhang[3✉], Lan Wang[4✉], Xun Gui[1✉]

Affiliations:
[1] Mabwell (Shanghai) Bioscience Co., Ltd., Shanghai 201210, China.
[2] Beijing Kohnoor Science & Technology Co., Ltd., Beijing 102206, China.
[3] School of Life Sciences, Anhui University, Hefei 230601, China.
[4] Key Laboratory of the Ministry of Health for Research on Quality and Standardization of Biotech Products, National Institutes for Food and Drug Control, Beijing 100050, China.
[5] Ludwig Cancer Research, Nuffield Department of Medicine, University of Oxford, Oxford OX37DQ, United Kingdom.
[*] These authors contributed equally: Shuang Wang, Junchao Wang, Xiaojuan Yu, Wen Jiang, Shuo Chen, Rongjuan Wang.
✉email: Jinchao.zhang@mabwell.com; zhmin07@ahu.edu.cn; wanglan@nifdc.org.cn; xun.gui@mabwell.com

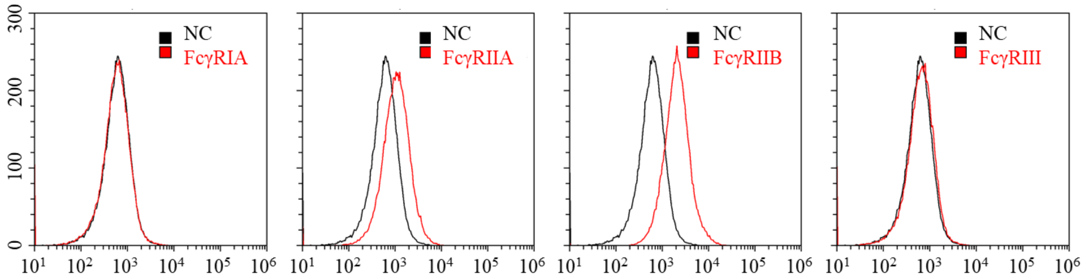

Supplementary Fig. 1. Expression profile of FcγRs on Daudi cells was determined by FACS. "NC" means isotype control.

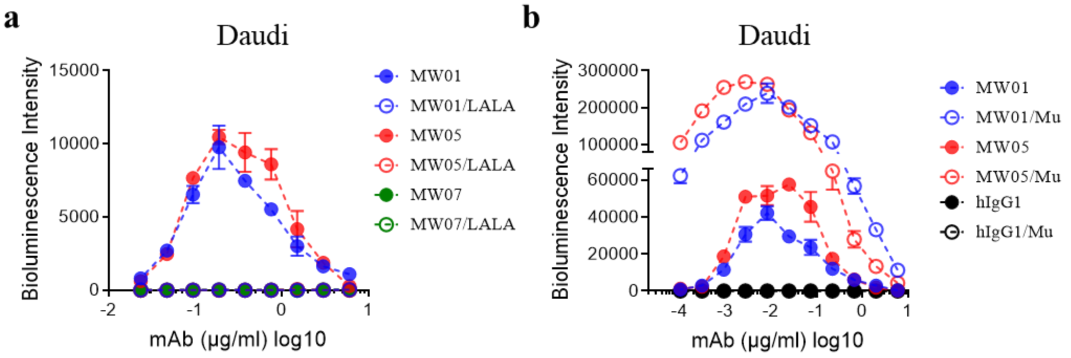

Supplementary Fig. 2. ADE of SARS-CoV-2 pseudoviral infection on Daudi cells. **a** Comparison of ADE activity mediated by wild-type and LALA mutated human IgG1 mAbs on Daudi cells. **b** ADE activity of wild-type and Fc region mutated human IgG1 mAbs on Daudi cells. "Mu" means G237D, P238D, P271G and A330R combined mutation on human IgG1 mAb, which increases the binding affinity to human FcγRIIB.

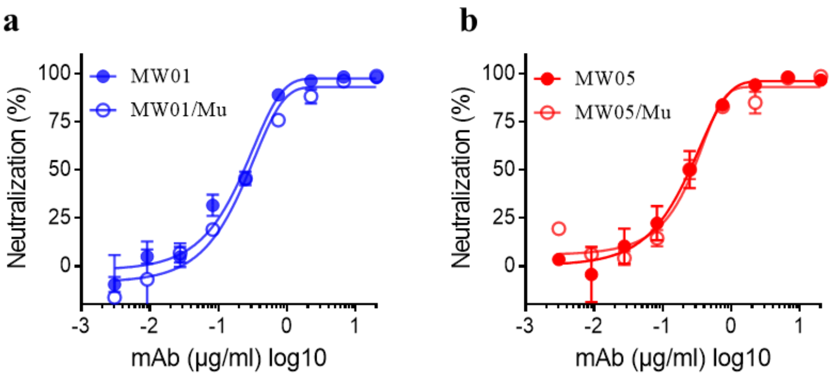

Supplementary Fig. 3. SARS-CoV-2 neutralization potencies of wild-type and mutated mAbs. a Neutralization potencies of MW01 and MW01/Mu were measured on SARS-CoV-2 pseudovirus system. **b** Neutralization potencies of MW05 and MW05/Mu were measured on SARS-CoV-2 pseudovirus system. "Mu" means G237D, P238D, P271G and A330R combined mutation on human IgG1 mAb.

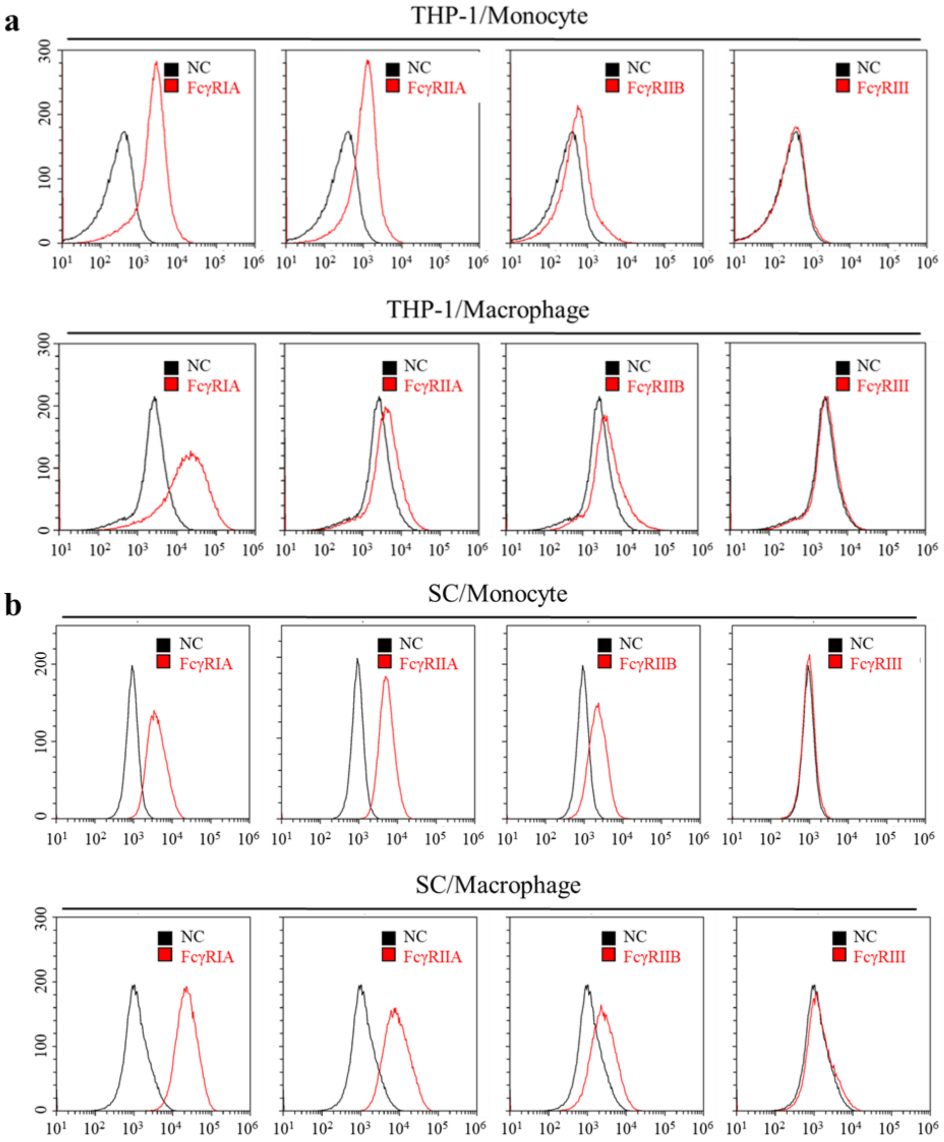

Supplementary Fig. 4. The expression level of FcγRs were checked by FACS. a The expression profile of FcγRs on THP-1 and THP-1differentiated macrophages. **b** The expression profile of FcγRs on SC and SC differentiated macrophages.

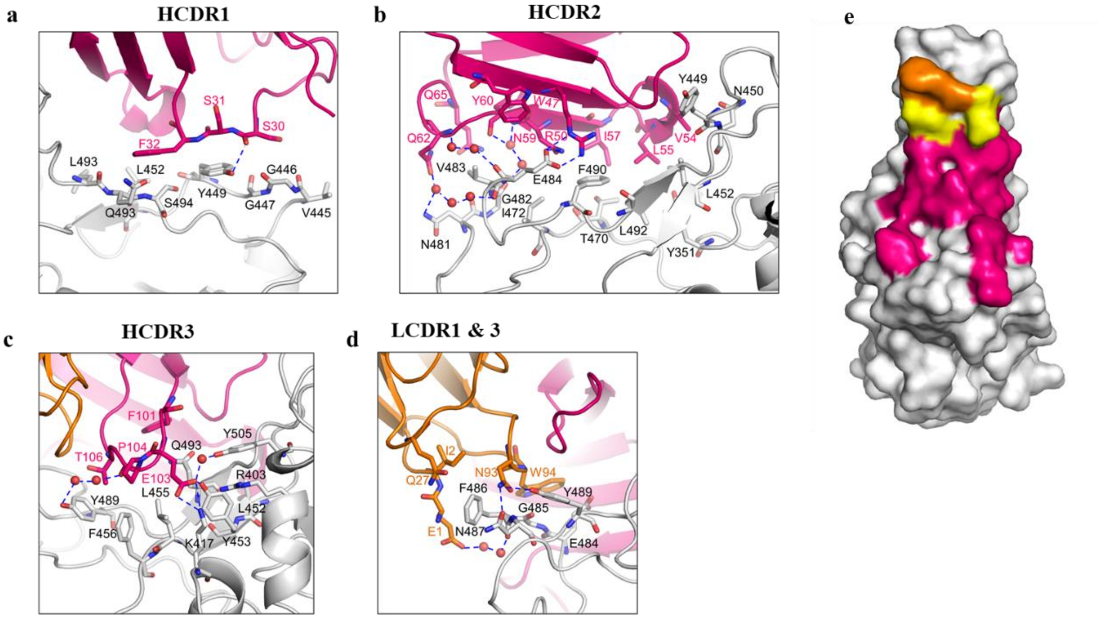

Supplementary Fig. 5. Structure analysis of MW01 Fab and SARS-CoV-2 RBD complex. a, b The detailed interactions between SARS-CoV-2 RBD and MW01 CDR loops of heavy chain. **c, d** The detailed interactions between SARS-CoV-2 RBD and MW01 CDR loops of light chain. The involved residues are shown as sticks with the same colors in Fig. 2A. The hydrogen bond networks are shown as red spheres. **e** The epitope of MW01 are shown in surface representation. The residues contacted by heavy chain, light chain, and both chains were colored in hot pink, red and yellow, respectively.

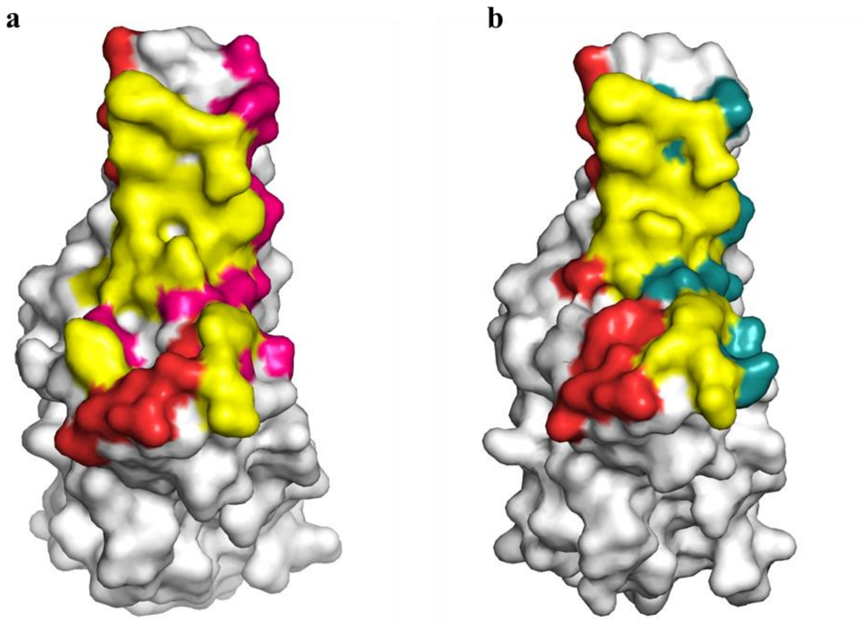

Supplementary Fig. 6. Competitive binding of hACE2 with MW01 and MW05. a The MW01 and hACE2 binding surface on SARS-CoV-2 RBD. The residues contacting MW01 and hACE2 are colored in hot pink and red, respectively, the residues contacting both are colored in yellow. **b** The MW05 and hACE2 binding surface on SARS-CoV-2 RBD. The residues contacting MW05 and hACE2 are colored in dark green and red, respectively, the residues contacting both are colored in yellow.

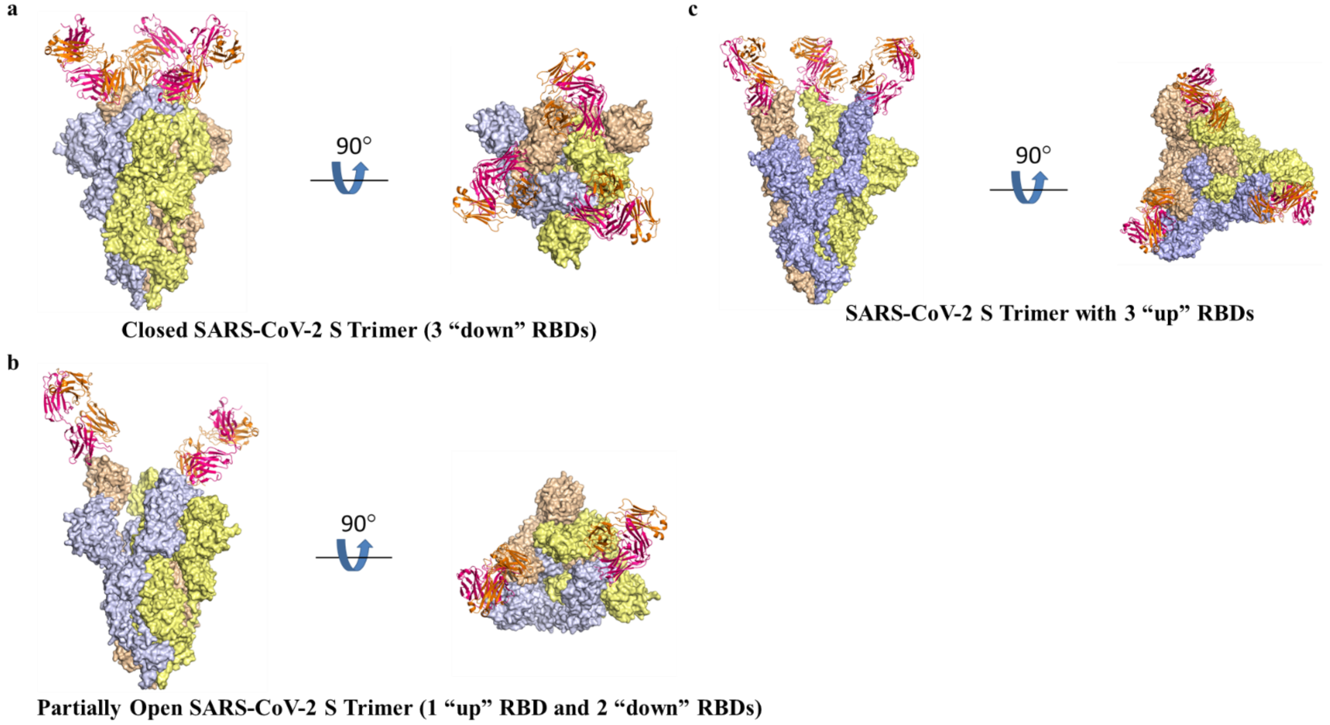

Supplementary Fig. 7. Models of MW01 Fab bound to SARS-CoV-2 S trimer. a Superimposition of MW01 Fab to closed SARS-CoV-2 S trimer (PDB code: 6VXX). Three MW01 Fabs are superposed to three RBDs in the closed SARS-CoV-2 S trimer. **b** Superimposition of MW01 Fab to partially open SARS-CoV-2 S trimer (PDB code: 6VYB). One MW01 Fab is superposed to the “open” RBD, and one MW01 Fab is superposed to one of the “down” RBDs in the partially open SARS-CoV-2 S trimer. Superimposition of MW01 Fab to the other “down” RBD will introduce clashes (not shown in this figure). The heavy chain and light chain of MW01 Fab are shown as hot pink and orange cartoons, respectively. The three chains of SARS-CoV-2 S trimer are shown as wheat, gray and yellow surface. **c** Superimposition of MW01 Fab to SARS-CoV-2 S trimer with 3 “up” RBDs (PDB code: 7A98). Three MW01 Fabs are superposed to three “up” RBDs in SARS-CoV-2 trimer.

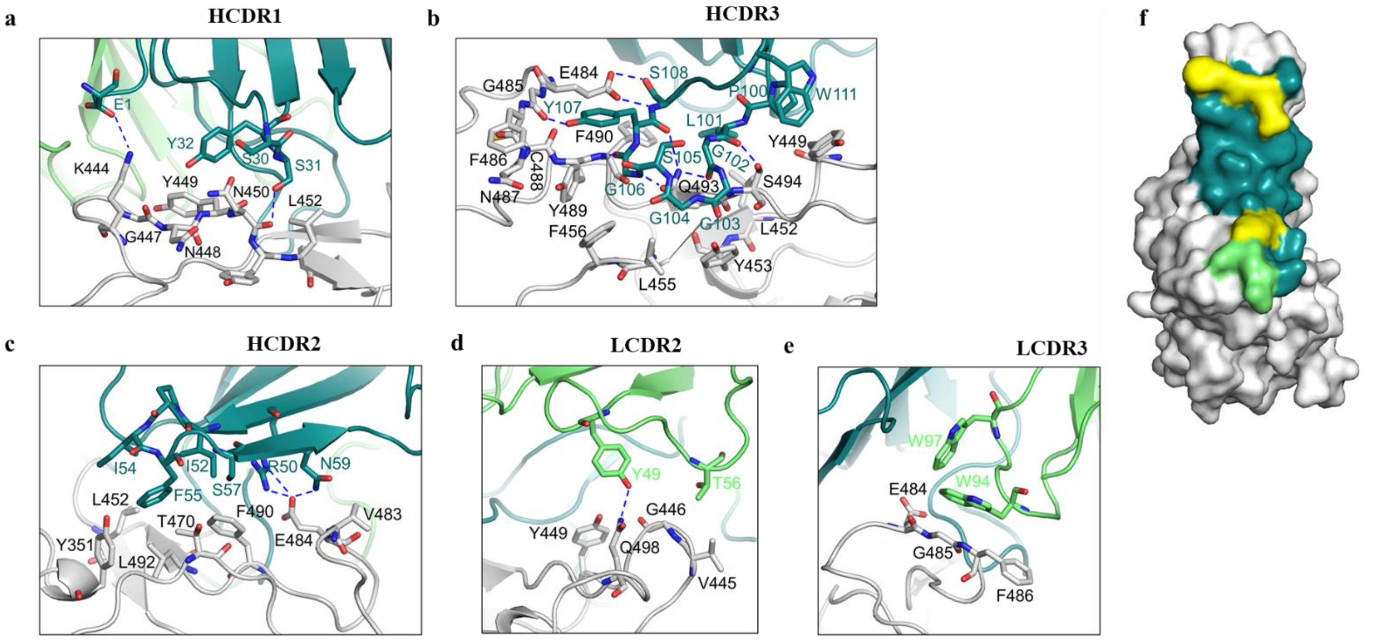

Supplementary Fig. 8. Structure analysis of MW05 Fab and SARS-CoV-2 RBD complex. a-c The detailed interactions between SARS-CoV-2 RBD and MW05 CDR loops of heavy chain. **d-e** The detailed interactions between SARS-CoV-2 RBD and MW05 CDR loops of light chain. The involved residues are shown as sticks with the same colors in Fig. 2A. The hydrogen bond networks are shown as red spheres. **f** The epitope of MW05 are shown in surface representation. The residues contacted by heavy chain, light chain, and both chains were colored in dark green, light green and yellow, respectively.

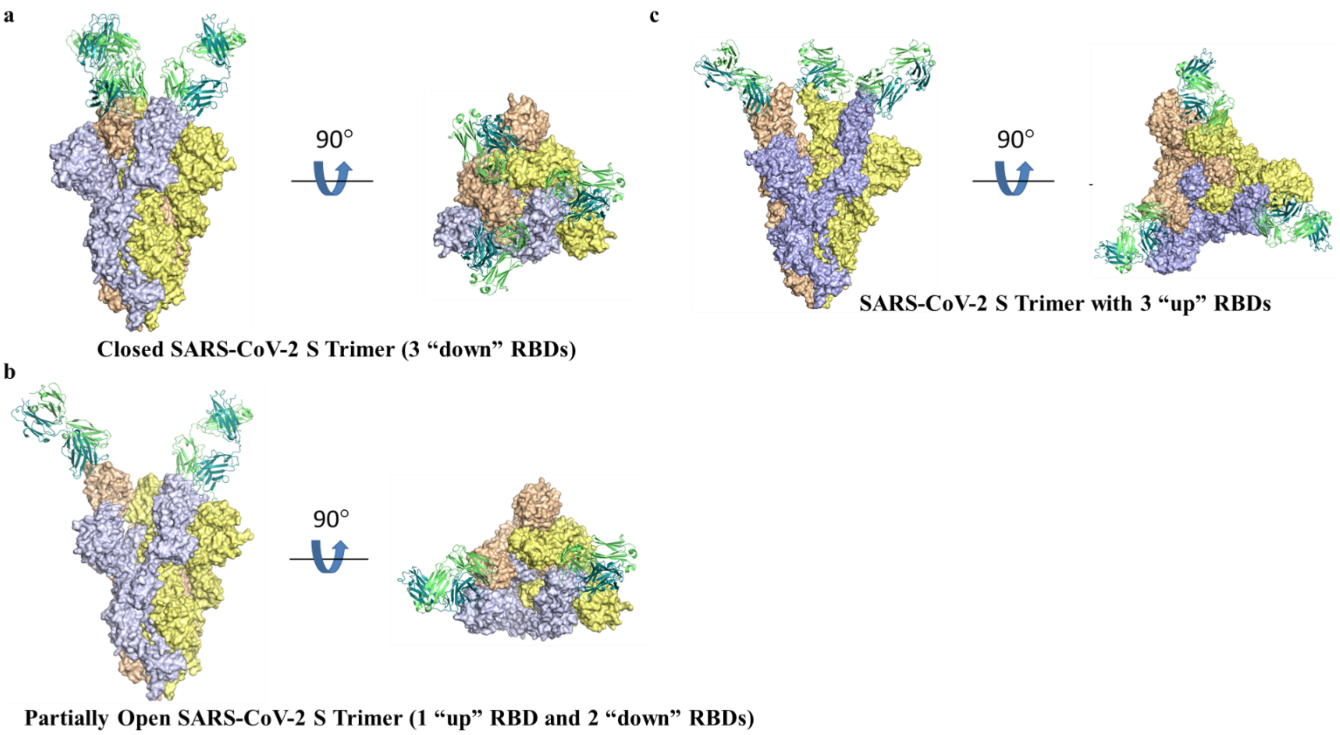

Supplementary Fig. 9. Models of MW05 Fab bound to SARS-CoV-2 S trimer. a Superimposition of MW05 Fab to closed SARS-CoV-2 S trimer (PDB code: 6VXX). Three MW01 Fabs are superposed to three RBD in the closed SARS-CoV-2 S trimer. **b** Superimposition of MW05 Fab to partially open SARS-CoV-2 S trimer (PDB code: 6VYB). One MW05 Fab is superposed to the “open” RBD, and one MW05 Fab is superposed to one of the “down” RBDs in the partially open SARS-CoV-2 S trimer. Superimposition of MW05 Fab to the other “down” RBD will introduce clashes (not shown in this figure). The heavy chain and light chain of MW05 Fab are shown as dark green and light green cartoons, respectively. The three chains of SARS-CoV-2 S trimer are shown as wheat, gray and yellow surface. **c** Superimposition of MW05 Fab to SARS-CoV-2 S trimer with 3 “up” RBDs (PDB code: 7A98). Three MW05 Fabs are superposed to three “up” RBDs in SARS-CoV-2 trimer.

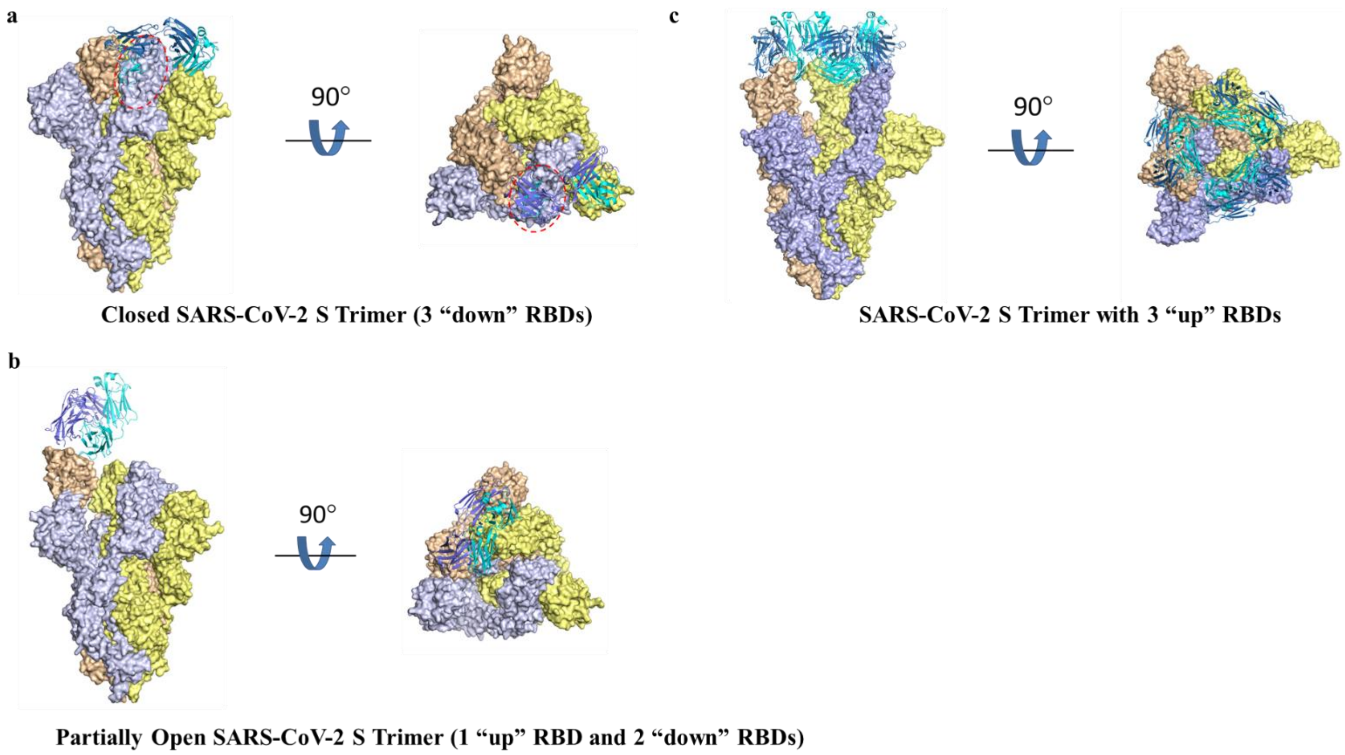

Supplementary Fig. 10. Models of MW07 Fab bound to SARS-CoV-2 S trimer. a Superimposition of MW07 Fab to closed SARS-CoV-2 S trimer (PDB code: 6VXX). The binding of MW07 Fab with one RBD (wheat) introduces clashes (enclosed with red dashed cycle) with another RBD (gray). **b** Superimposition of MW07 Fab to partially open SARS-CoV-2 S trimer (PDB code: 6VYB). MW07 Fab can only be superposed to the “open” RBD with no clashes. **c** Superimposition of MW07 Fab to SARS-CoV-2 S trimer with 3 “up” RBDs (PDB code: 7A98). Three MW07 Fabs are superposed to three “up” RBDs in SARS-CoV-2 trimer.

a

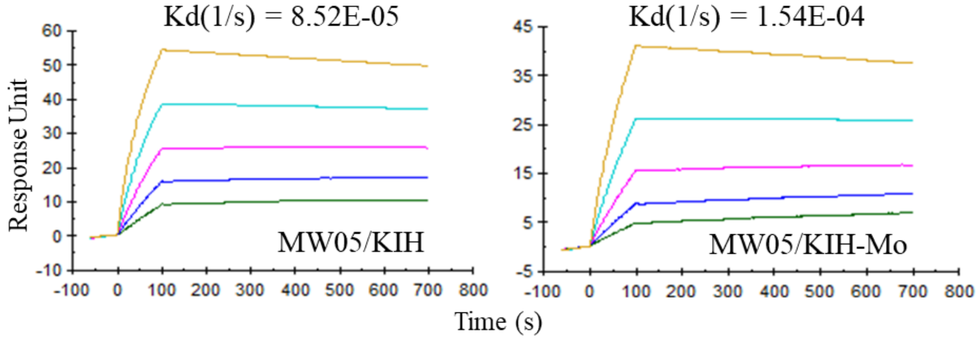

b

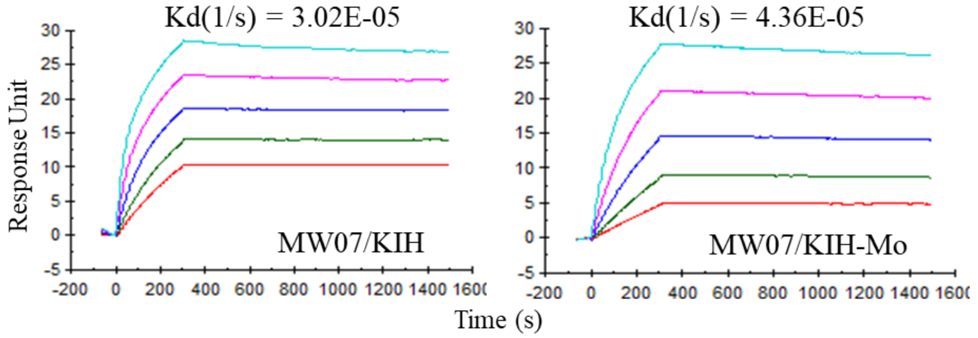

Supplementary Fig. 11. Measurement of dissociation signals of bivalent and monovalent mAbs. NTA biosensor chip was used to capture SARS-CoV-2 S trimer on BIAcore S200 system. 2-fold serially diluted monovalent and bivalent MW05 (**a**) and MW07 (**b**) starting from 100 nM were then flowed over the chip surface. "KIH" means knob-in-hole format bivalent mAb. "KIH-Mo" means knob-in-hole format monovalent mAb.

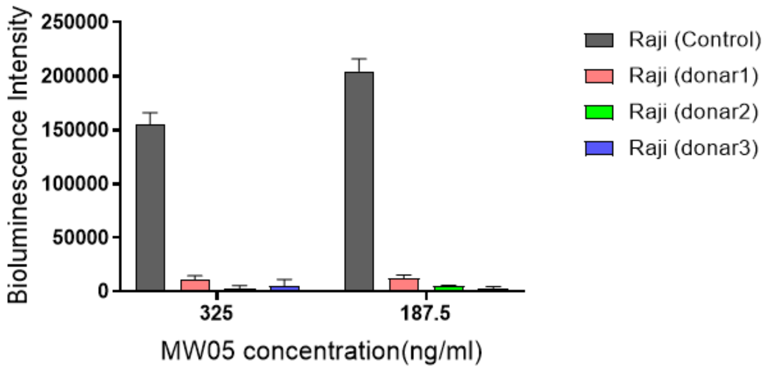

Supplementary Fig. 12. **ADE mediated by MW05 on Raji cell treated with convalescent serum from COVID-19 patients.** Comparison of ADE effects of MW05 on Raji cells treated with serum from 3 COVID-19 patients. The concentrations of MW05 used in this assay are 325 ng/ml and 187.5 ng/ml, which are the highest ADE signal related concentrations. Before incubating with the mixture of MW05 and pseudo-virus, Raji cells were incubated with 10-fold diluted serum for 1h.

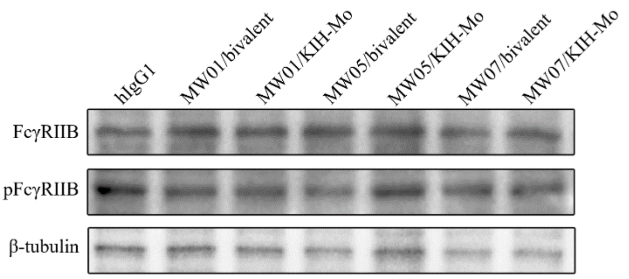

Supplementary Fig. 13. FcγRIIB phosphorylation in Raji cells were determined by WB. Raji cells were treated with the complex of SARS-CoV-2 pseudovirus together with MW01, MW05 or MW07 (the same condition as the ADE assays). Concentration used in this assay is 375 ng/ml, which is a high ADE signal related mAb concentration.

Supplementary Table 1. Contacts of SARS-CoV-2 RBD with MW01 and MW05

SARS-CoV-2 RBD	MW01		MW05	
	Heavy chain	Light chain	Heavy chain	Light chain
Y351	L55		I54, F55	
R403	E103			
K417	E103			
K444			E1	
V445	S30			T56
G446	S30			Y49, T56
G447	S30		Y32	
N448			Y32	
Y449	S30, S31, F32, V54		Y32, P100, W111	Y49
N450	V54		S30, S31, Y32	
L452	F32, V54, L55		S31, F55, L101	
Y453	E103		G103	
L455	E103		G104	
F456	P104, T106		G106	
T470	I57		F55, S57	
I472	N59			
N481	Q62			
G482	Y60, Q65			
V483	Y60, Q62, Q65		N59	
E484	W47, R50, N59, Q62	W94	R50, N59, S108	W94, W97
G485		W94	Y107	W94
F486		E1, I2, Q27, N93, W94	Y107	W94
N487		N93	Y107	
C488			Y107	
Y489	P104, T106	N93	G106, Y107	
F490	R50, I52, L55, I57, N59		I52, S57, G106, S108	
L492	F32, L55		F55	
Q493	F32, F101		L101, G102, G104, S105, G106, Y107	
S494	F32		L101, G102	
Q498				Y49
Y505	E103			
